# To Remove Color of Iodine and Permanganate of Potassium

**Published:** 1898-04

**Authors:** 


### To Remove Color of Iodine and Permanganate of Potassium.

Answering the inquiry for colorless iodine, I would give the following: Add a saturated sol. of hyposulphite of soda to tincture of iodine slowly, and stir till all the color disappears. No doubt in decolorizing in any manner there is a new chemical composition, but whether this affects the action of the original tincture or not is a question. To remove stains of tincture of iodine from clothing, skin, etc., the hyposulphite of course acts beautifully.

For removing the stains of permanganate of potassium from the hands or clothing, peroxide of hydrogen acts like magic.—*Bret Black, M. D., in Medical World.*

